# Non-Contact Multiscale Analysis of a DPP 3D-Printed Injection Die for Investment Casting

**DOI:** 10.3390/ma14226758

**Published:** 2021-11-09

**Authors:** Arkadiusz Kroma, Michał Mendak, Michał Jakubowicz, Bartosz Gapiński, Paweł Popielarski

**Affiliations:** 1 Division of Foundry and Plastic Working, Institute of Materials Technology, Faculty of Mechanical Engineering, Poznan University of Technology, 60-965 Poznan, Poland; pawel.popielarski@put.poznan.pl; 2 Division of Metrology and Measurement Systems, Institute of Mechanical Technology, Poznan University of Technology, 60-965 Poznan, Poland; michal.jakubowicz@put.poznan.pl (M.J.); bartosz.gapinski@put.poznan.pl (B.G.)

**Keywords:** DPP, 3D printing, X-CT, focus variation microscopy, structured blue-light scanner, investment casting

## Abstract

The investment casting method supported with 3D-printing technology, allows the production of unit castings or prototypes with properties most similar to those of final products. Due to the complexity of the process, it is very important to control the dimensions in the initial stages of the process. This paper presents a comparison of non-contact measurement systems applied for testing of photopolymer 3D-printed injection die used in investment casting. Due to the required high quality of the surface parameters, the authors decided to use the DPP (Daylight Polymer Printing) 3D-printing technology to produce an analyzed injection die. The X-ray CT, Structured blue-light scanner and focus variation microscope measurement techniques were used to avoid any additional damages to the injection die that may arise during the measurement. The main objective of the research was to analyze the possibility of using non-contact measurement systems as a tool for analyzing the quality of the surface of a 3D-printed injection die. Dimensional accuracy analysis, form and position deviations, defect detection, and comparison with a CAD model were carried out.

## 1. Introduction

The competitiveness of enterprises is very often associated with the implementation of the newest manufacturing techniques and corresponding control techniques, which translates into easier and faster satisfaction of the increase in customer requirements for manufactured products. At the same time, the development of more and more complex products entails the necessity to produce prototypes whose properties will correspond to the production (final) part as much as possible [1,2]. One of the techniques allowing for small-lot and accurate production of complex-shaped castings is the investment casting method [3], both in the traditional form and in the version based on 3D-printing technology [4].

The development of 3D-printing technologies made it possible to design and manufacture elements with geometries impossible to obtain with traditional methods, while meeting the requirements of dimensional, shape, and position tolerances. These methods are used primarily in the field of rapid prototyping and tooling. In the case of the investment casting method discussed by the authors, additive technique is most often used as a substitute for the traditional method of producing wax models (wax patterns) [5], which in the so-called classical method are formed by injecting a liquid or semiliquid wax mixture into a reusable form called an injection die. These injection dies can be made of metal alloys (mass production) or elastomers such as rubber or silicone (unit/small series production) [2]. In mass production, due to the high rate of heat conduction, which directly increases a company’s efficiency, aluminum and copper alloys are primarily used [4]. In addition, the use of such materials shortens the solidification time, as a result of which there is no excessive crystal growth [6], which improves the mechanical properties of the wax models. Rubbers and silicones are used primarily in unit and prototype production of castings [2], where the time needed to make a wax model and its mechanical properties are not the most important factors in the production process. Since the quality of injection dies is reflected in the quality of the models, which directly affects the quality of the castings, it is very important to verify the real shape of obtained geometry at the stage of production start.

The application of 3D-printing technology in the case of unit production allows the omission the time-consuming and often costly stage of producing the necessary equipment [7], which can be seen in the case of the plastics industry. It is becoming more and more common to perform short, pilot production series (several—over a dozen elements) with the use of replaceable, 3D-printed inserts for injection molds [8] made of a special material resistant to high temperature [9,10]. The leader in the field of application of 3D-printed injection mold elements is Stratasys Ltd., which uses its patented PolyJet photopolymer 3D-printing technology (minimal layer thickness is 16 µm). This technology is characterized by high quality of products, while being one of the most expensive currently existing 3D-printing techniques [11]. In addition, direct printing of casting patterns allows for quick and cheap modification of products, which is crucial during the production of prototypes [10]. In this method, the casting models are used to make a disposable casting mold, which is then heat treated to improve its properties and allow a mold cavity to be formed by removing disposable wax patterns. Therefore, wax models should be characterized by low roughness, good geometry reproduction, and ash-free burnout [4,12]. Only a few materials available on the market are subject to ash-free burnout, e.g., HIPS, ABS, photopolymer casting resin (ash content TGA 0.0–0.1%) [13]. The most commonly used 3D-printing techniques are: FDM (fused deposition modelling), SLA (stereolithography) [1], SLS (selective laser sintering) [14], DPP (daylight polymer printing) [15] and DLP (digital light processing) [16]. 

**Figure 1:**
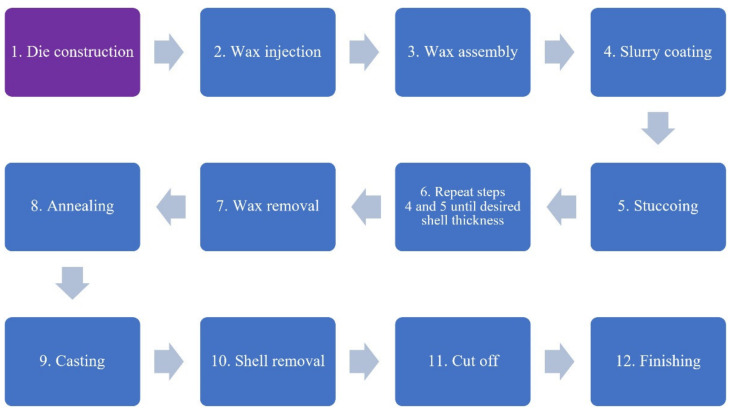
Schematic view of the investment casting process with the analyzed production stage marked with purple color.

Very often, the production of prototype castings takes place in gypsum molds. This is due to, inter alia, availability, price, and ease of making compared to classic molds based on hydrolyzed ethyl silicate [5,6]. Unfortunately, due to the limited susceptibility of the gypsum mold and the improper choice of materials, the models increase their volume during annealing, permanently damaging the mold cavity [17]. The simplest solution to this situation is the use of flexible gypsum, which is characterized by increased flexibility, thanks to which, in the first stage of the annealing process, the mold is not permanently damaged. This procedure results in an increase in the costs of producing a casting mold and the need to store additional molding material (e.g., flexible gypsum), which indirectly extends the implementation time of new products many times. The block diagram of the investment casting process with particular emphasis on the analyzed production stage (purple color) is shown in Figure 1.

However, the use of 3D-printed casting models is not always possible and economically justified. The need to have a qualified employee, a 3D printer, the number of different materials necessary to start the process, and the profitability of this method only in the case of unit production, can be a significant barrier for many foundries. Therefore, in order to avoid costly investment outlays, the authors decided to propose a different, proprietary approach to the described issue, consisting of direct 3D printing of the injection die using DPP technology.

The application of 3D-printed injection dies to produce wax patterns is a similar process to that used in the plastics industry. However, due to the properties of the injected material, it requires a different approach. Contrary to plastics, the injection temperature of the wax mixture, depending on its type and manufacturer, ranges, e.g., from 71 to 79 ℃ [18]. However, it is common to encounter a situation where the casting machine applies a wax mixture at temperatures lower than those recommended by the manufacturers. The technological process carried out in this way, involving the use of a semi-liquid wax mixture, can be compared to the extrusion process, and it results from the desire to avoid excessive shrinkage of the solidifying wax mixture and to accelerate the process of producing wax patterns (shortening the solidification time). In addition, the maximum temperature level of the injected wax mixture allows the use of a much larger number of materials for the production of wax injection dies than in the case of plastic injection, due to the lack of the need to meet the requirements for resistance to high temperature. The selection of materials depends primarily on the possibility of their use in the 3D-printing process, price, availability, and glass transition temperature, which determines the increase in the viscosity of the substance and must be higher than the temperature of the wax mixture (it has a direct impact on the durability of the injection die). Thus far, the application of 3D-printing technology to produce wax injection dies, and their analysis, have not been widely discussed in leading scientific journals. In 2020, the articles [19,20] presented an analysis of the surface topography of injection die made with PolyJet technology using a profilometer. The authors also analyzed the relationship between the surface parameters and the number of wax injections. Therefore, it was decided to present the results of the analysis of the possibility of using non-contact technologies for wax injection dies measurements, conducted in parallel at the same time, which, according to the authors of this article, due to their non-destructive nature, are a much better solution to contact technologies.

Due to the characteristics of the investment casting process, the key issue is to keep the casting models intact at every stage of the preparation of the casting mold. Even the smallest error in the form of damage or production fault of injection die will result in the wrong shape of the model, consequently producing a defective casting [5]. The quality of the 3D-printed parts is influenced by many parameters, such as the orientation of the model in the printer’s working space [21], the thickness of a single layer [22], printing technology and material [23], and number and type of supports, or (in the case of FDM) type of infill [24]. Therefore, in order to verify the quality of 3D printing, it is necessary to carry out a geometric analysis of the injection die. This analysis can be performed in two different approaches:

- Indirect—geometric analysis of the wax models formed in the injection die;
- Direct—geometric analysis of the 3D-printed injection die.

The indirect measurement of the obtained injection die can be performed in a much easier way than the direct measurement, because permanent damage to the wax model does not influence the surface or dimensions of the die and will only result in the necessity of production of another wax model. In this situation, it is possible to use measurement methods such as contact methods (e.g., profilometry, measurements on a CMM). Model geometry analysis, in the case of investment casting technology, allows obtaining information directly related to the future casting. Unfortunately it does not provide any information about the injection die itself. The variability of the injection process of the liquid or semi-liquid wax mixture into the injection die may result in a noticeable variation in the geometry of the wax models (a change in the injection pressure, or the wax itself may result in a change in the shrinkage of the models) [25,26]. As a result, the obtained initial analysis results may be highly variable over time.

Direct measurement of 3D-printed injection dies requires the use of the most modern non-contact measurement techniques (e.g., X-ray CT, structured blue-light scanner), which do not damage the measured surface. Such a measurement is characterized by precise information about the analyzed die, which allows selection of the parameters of forming wax models faster and more accurately. It is not without significance that, as a result of contactless geometric analysis, the injection die is not damaged and can be used immediately after the measurements [27,28].

The obvious fact is that the purchase of the proper measuring equipment is an expensive investment. In the case of foundries that deal with small-scale production of prototype elements, the stability of the process (both for the production of patterns and castings) is one of the most important factors of work. Contactless measurements can be used at the initial stage of the implementation of new casting tooling as the base values, which will later serve as references to the results obtained with the use of contact (indirect) analyses.

## 2. Materials and Methods

### 2.1. Model and Injection Die

**Figure 2:**
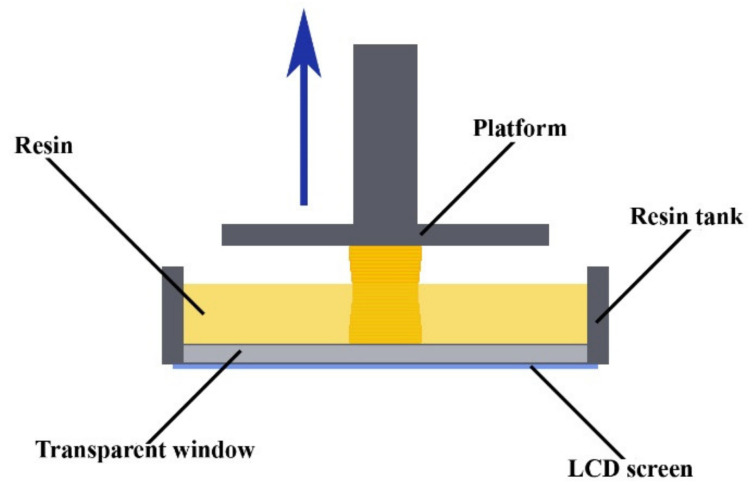
Schematic view of DPP process.

Due to the characteristics of the investment casting process, wax injection dies have high dimensional and geometric accuracy, as well as low surface roughness. Therefore, to produce the analyzed sample, the authors decided to use the photopolymer DPP 3D-printing technology, which is characterized by low minimal layer thickness (25 µm) and provides lots of different materials with an affordable price (USD 50–125 per liter). This 3D-printing technology uses light from LCD screens to create 3D-printed parts by curing liquid daylight photopolymer resin layer by layer. The diagram of the technology is shown in Figure 2.

The DPP technology allows the use of cheaper materials than those used in other photopolymer 3D-printing technologies and allows for simultaneous exposure of the entire cross-section of the element, which is not possible in the case of point technique such as SLA 3D printing [15]. At the same time, it should be remembered that each 3D printer, apart from the minimum thickness of a single layer in the Z axis, also has an XY resolution. In the case of parts printed on the basis of a light source, which is an LCD screen, the key parameter is the size of a single pixel, because it determines the correct mapping of the surface curvatures. Moreover, one of the software methods of improving the surface quality of 3D-printed elements is the anti-aliasing function, which consists in smoothing curves by color intensity [29]. 

**Figure 3:**
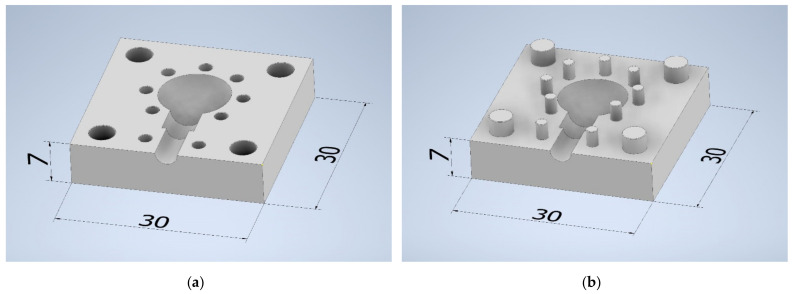
The 3D model of the designed injection die: (**a**) female part; (**b**) male part.

The main element of the model (Figure 3) designed in Autodesk Inventor Professional 2020 (Autodesk Inc., Mill Valley, CA, United States of America) was a spherical part with a diameter of 11.18 mm, which was used due to the uniformity of shrinkage during solidification of wax patterns and castings. Moreover, the geometry consists of a cylindrical part with a length and diameter of 5 mm and a gating system 7 mm long and 3.5 mm in diameter. The injection die consists of two parts (male and female part).

For the research, the Liquid Crystal HR (Photocentric Ltd., Cambridge, UK) 3D printer was used, which is characterized by a 9.7” LCD screen with a resolution of 2048 × 1536 pixels (196 mm × 147 mm), XY resolution of 97 µm, and compatibility with Creation Workshop slicer software v. 1.0.0.75 (Envision Labs, Indianapolis, IN, United States of America). The analyzed model was placed perpendicularly to the platform, the Photocentric Flexible Resin [30] was used, the thickness of a single layer was 50 µm, and the total printing time took 7 h. The 3D-printed injection die was cleaned with the Photocentric Resin Cleaner, then placed in clear water and 15 min of exposure with a 36 W, 405 nm UV lamp was carried out in order to homogenize the strength parameters. The injection die prepared in this way was left to dry freely overnight.

### 2.2. Measurement Technologies and Measurement Strategy

Since the main objective of this study was to evaluate several measurement systems and their ability to detect and characterize defects on the surface, as well as their performance in three-dimensional measurement, choosing the right measurement technology to measure the 3D-printed part was essential to provide results within the shortest period of time, while assuring the sufficient accuracy of the measurement. Metrology for additive techniques, while still under development, has already helped to gather a reasonable amount of knowledge [28,31,32] and provided valuable information about performance of already existing technologies [33,34,35]. Some studies compared the performance of contact and non-contact measurement technologies in terms of their accuracy and measurement uncertainty [36,37,38]. Using this knowledge, one must consider several material characteristics:

- Material’s toughness and pliability—characteristics essential when considering tactile measurement. To some extent, a contact stylus may scratch and deform the surface, rendering the results unreliable;
- Transparency and light dispersion—a limiting factor when using optical technologies. Transparency limits the use of focus- or contrast-based methods (such as Focus Variation, focus stacking, etc.), and light dispersion decreases either contrast (in image-based measurement) or signal strength;
- Varying surface reflectivity—this factor is especially present in powder-based technologies. However, complex shapes, that are possible to achieve in additive manufacturing, can render an adequate exposure adjustment problematic.

**Figure 4:**
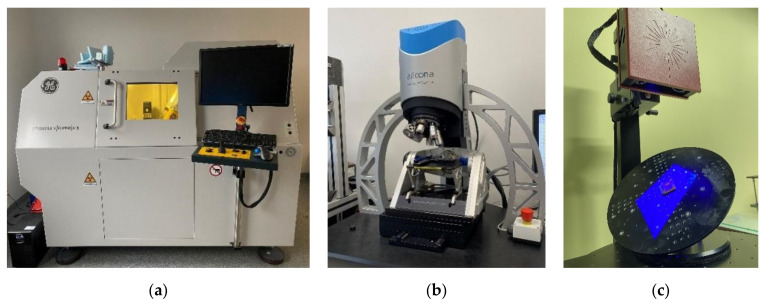
Measurement devices used in the study: (**a**) X-ray CT; (**b**) focus variation microscope; (**c**) structured blue-light scanner.

The main limitation regarding the choice of suitable measurement technologies was the characteristic of the die’s material, which was slightly pliable. For this reason, the researchers could not use any tactile method (neither a CMM nor a profilometer) as it would either produce unreliable results or scratch the surface [39]. This limited the array of usable technologies to only non-contact methods. The second limitation was the mold’s geometry, i.e., the small radius on the edge of the sphere’s cavity, and its small dimensions in general. A suitable measurement solution would have a sufficient resolution to reliably measure these details. Other challenging geometrical features were relatively high slope angles, and long measurement distance (along the optical axis) from the parting plane to the bottom of the spherical cavity, which disqualified measurement techniques such as CSI or confocal microscopy and profilometry [40]. A suitable device should be able to fully measure the element with as few single measurements as possible, and to detect microscale defects [41,42,43]. Several studies have addressed the need for comparison of optical methods [44,45]. Measurement strategies are described in separate chapters. The researchers used manufacturers’ native software to collect data and export it to an STL file. GOM Inspect 2018 (GOM GmbH, Braunschweig, Germany) software was used for GD&T analysis. Three measurement techniques were selected for this evaluation: an X-ray CT Waygate Technologies, Wunstorf, Germany), a focus variation microscope (Alicona Imaging GmbH. Gratz, Austria), and a structured blue-light optical scanner (GOM GmbH, Braunschweig, Germany) (Figure 4a–c).

#### 2.2.1. X-ray CT

Researchers used, presented in Figure 4a, the micro-CT device Phoenix V|tome|x S240 (Waygate Technologies, Wunstorf, Germany). The device consists of a transmission X-ray tube emitting a conical X-ray beam, rotating table where the tested sample is placed and the detector recording X-ray images corresponding to successive angular positions of the parts.

X-ray is an electromagnetic radiation generated in an event of decelerating a charged particle, i.e., an electron, when it deflects (decelerates) on another charged particle, i.e., atomic nucleus. An electron’s kinetic energy is converted into electromagnetic radiation. In X-ray measurement devices, this radiation is created inside a vacuum tube, where electrons are accelerated from a source (typically a tungsten filament) onto a target, thus emitting a cone-shaped beam of electromagnetic radiation. An element, which is positioned on the beam’s path, absorbs some of the photons’ energy (depending on the material’s density and thickness), which then hits the detector behind it. The detector utilizes a scintillator array to convert X-ray radiation into visible-light quanta, which are then detected by the CCD sensor. The intensity of the signal acquired by the latter sensor is expressed as a value on a grayscale, thus creating an X-ray image. The device takes multiple exposures (scans) while rotating the sample, which allows for its later 3D reconstruction.

**Figure 5:**
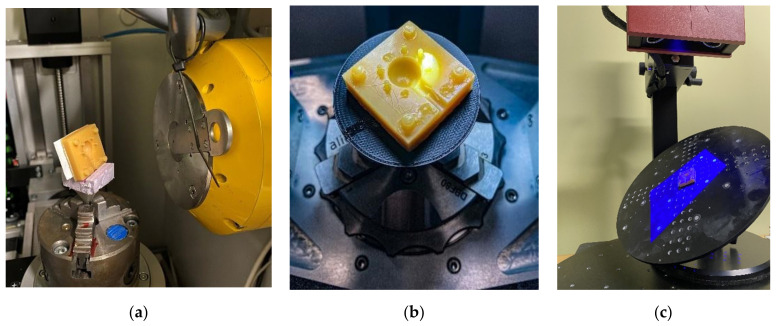
The element’s fixture in consecutive measurement systems: (**a**) X-ray CT, (**b**) focus variation microscope, (**c**) structured blue-light scanner.

The specimen was placed on a sturdy cylinder using an extruded Styrofoam adapter (Figure 5a), ensuring that no specimen displacement would occur during the measurement. The stage was moved as close to the x-ray tube as possible, limited only by the specimen’s dimensions and allowable power level. The power limit is crucial to the quality of the measurement, since, with an increase in power, a focal spot of the cone beam is enlarged. This affects the “shadow” of the part, rendering the image blurrier. Prior to the series of measurements, the detector was calibrated for the chosen beam settings. A total of five measurements were performed, with minimum stoppage time between the consecutive measurements. An 8 mm calibration ballbar standard was used to calibrate the device’s dimensional accuracy in the measurement position. Measurement settings are presented in the Table 1.

The voxel size was dictated by the need of performing the scan within the single Field of View of the device in order to both avoid any merging errors and to limit the unnecessary prolongation of measurement duration. The number of projections was set according to both manufacturer’s guidelines and research results [46]. Since the sphere’s nominal diameter was equal to 11.18 mm, its width on an X-ray image would be around 745 pixels. Other sources recommend to multiply an object’s width (in pixels) by 1.6 [47], which, in this case, resulted in the minimum number of projections being 1200. The researchers chose the value of 1500 projections to ensure strictest criteria throughout the measurement volume.

Surface determination was performed in Volume Graphics VGStudio software (Volume Graphics GmbH, Heidelberg, Germany), using standard ISO50 method. Volumetric data were converted to a surface mesh (STL) file with no simplification to the dataset. The use of X-ray CT in coordinate measurements has been investigated in other studies [48,49].

#### 2.2.2. Structured Blue-Light Scanner

The authors used the GOM Atos structured blue-light scanner system (Figure 4c) (GOM GmbH, Braunschweig, Germany). The scanner uses both triangulation and fringe-pattern projection to precisely measure even minute geometries within its resolution range. A drifting fringe pattern is projected onto a part and multiple exposures are taken and registered by two independent optical systems and then processed into a point cloud dataset. In order to accurately measure the parts, geometry reference points are placed in the measurement area. The operating principles of these techniques have been thoroughly described by many other researchers [27,50] and are widely in use, especially in the automotive and reverse-engineering industries. 

In this application, the printed mold was mounted onto a tilted, rotary table using an adhesive tape (Figure 5c). The tilt angle was set at 45 degrees and then the part was measured at eight, equally distributed positions around the rotary axis. Three additional measurements were performed in order to patch any non-measured areas.

#### 2.2.3. Focus Variation Microscope

In this study, an Alicona’s Infinite Focus G5 (Alicona Imaging GmbH. Gratz, Austria) device was used. Focus Variation is an areal measurement technology that utilizes microscope optics mounted on an ultra-precise vertical stage. Unlike the focus-stacking technique, which stitches in-focus points from several pictures, Focus Variation measures the discrete sampled signal of the “focus-value” between the neighboring pixels and an algorithm applies a curve-fitting function to estimate the Z-coordinate for maximum focus position [51]. An FVM device (Figure 4b) is capable of registering surface on slopes up to 87 degrees with nanoscale resolution along the optical axis. Lateral resolution is dependent on the objective used. Measurement set up parameters are listed in Table 2.

A 5× objective was used in order to, firstly, achieve a desired lateral resolution, and, secondly, shorten the measurement time. Coaxial illumination was used in polarized mode, due to varying intensity of reflected light. A total of six measurements were performed: four main, that captured ca. 90% of the cavity’s geometry, and two complementary measurements of the sphere’s deepest area (in Z axis) and flaking zone on the cylinder. The measured die was mounted on a 3D-printed conical adapter using a soft adhesive tape (Figure 5c).

**Figure 6:**
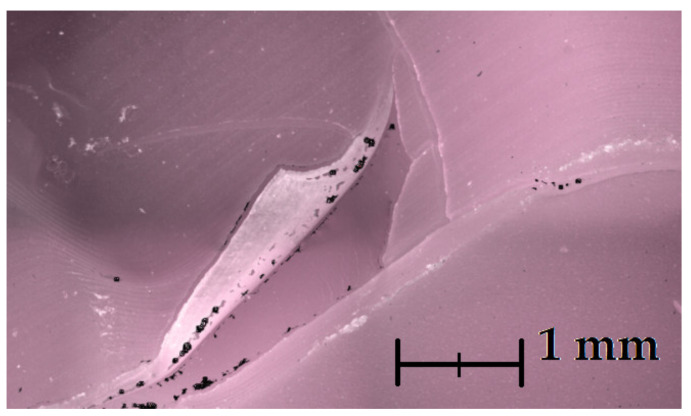
Image of a flaking site on the cylindrical section of the parts’ geometry created during the measurement with a focus variation microscope. A red hue is a side effect of using a polarized light source. Black spots are non-measured points.

A significant advantage of the FVM technology is that it also registers a true colored image of the sample. This allows one to visually inspect the element and assess its quality, as seen in Figure 6.

### 2.3. Dataset Evaluation

**Figure 7:**
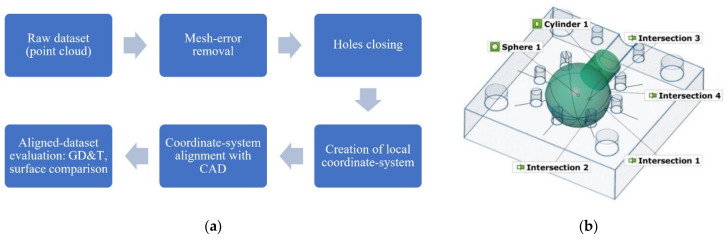
(**a**) Schematic view of the evaluation flow; (**b**) visualization of the measured geometries.

Each dataset was subjected to mesh-errors removal, and all holes within the measured area (a cavity consisting of a sphere and a cylinder) were closed using a smooth shape substitution based on neighboring points. Datasets from each measuring device were then subjected to an identical analysis scheme (Figure 7a) to determine deviations of dimensions, form, and position. Firstly, a local coordinate system based on geometric elements was created. The data for this system were: a parting plane, a cylinder, and a sphere’s center point, consecutively. The CAD model was also subjected to this procedure. Lastly, all datasets were aligned to the software’s Global Coordinate System and then exported as an STL file. This allowed for both surface data comparison to the CAD model, and to analyze the most significant geometrical features, as well as form and position deviations.

Each element, i.e., sphere, cylinder, circles, was created using an adequate software command, Create Fitting Element. The surface points for a given element were selected using an auto-expand function in the point selection menu. The elements were fitted using the Gaussian fitting method. Using this function allowed the researchers to automatically discard cracks and flaking, which would significantly alter the measurement results. Only regions of circular cross-sections on the cross-sections were selected manually. Due to the obvious printing error in the upper section of the sphere cavity, only the points below this artefact were selected for circle fitting. 

All geometrical and tolerance features were derived from the measured surface, using the Gaussian best-fit method. The “used points” setting was set to 3 sigma. Fitting elements used in geometric evaluation are presented in Figure 7b.

#### 2.3.1. Dimensional Accuracy

A significant characteristic regarding the wax-injection form is its dimensional accuracy. The injected wax is capable of highly accurate reproduction of the model geometry, which puts the burden of dimensional conformity on the die’s side. The following dimensions were checked in the tested model:

- Sphere’s diameter;
- Cylinder’s diameter;
- Circles’ diameter on each of the four, evenly distributed cross-sections.

**Figure 8:**
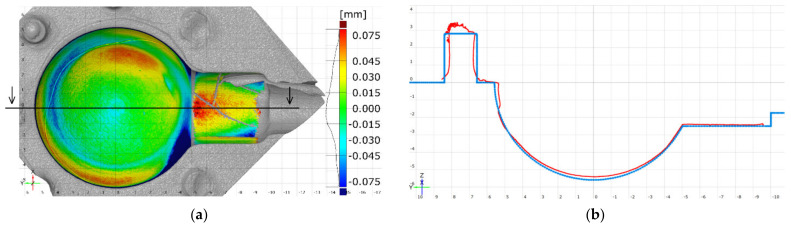
Printed element’s deviations: (**a**) from a fitted sphere and cylinder; (**b**) nominal (blue) and actual (red) cross-sections.

Both diameters are essential dimensions describing the examined mold. However, the researchers decided to evaluate the sphere’s radius along four, equally distributed planes pivoted around the Z axis. This would provide information on sphere-diameter variations in the main planes (Figure 8a,b).

#### 2.3.2. Form and Position Deviations

Another very important aspect of the 3D-printed parts correctness analysis is the extent of form and position deviations, and whether they do not exceed the tolerance limits set by the designer. In the case of the tested model, the following deviations were evaluated:

- Cylindricity;
- Roundness deviation of each cross-section to the fitted circles;
- Each fitted circle’s center-point position in relation to Plane Z (XY) in accordance with ISO 1101;
- Sphere’s center-point position in relation to Plane Z (XY) in accordance with ISO 1101.

The latter two are essential, since it is a two-part mold. Any deviation to the sphere’s position above the parting surface (Plane XY Datum) would result in a wax model deformation. As stated before, circular cross-sections were chosen over the standard sphericity inspection, in order to evaluate the printing process along the printers’ axes.

#### 2.3.3. Defect Detection

Researchers did not have access to software that was capable of automatically detecting surface defects, i.e., cracks and flaking. Therefore, only the measurement systems’ ability to visualize the defects was evaluated, based on the following criteria:

- Defect’s visibility on the dataset;
- System’s ability to measure both width and depth of the fractures;
- Visual quality of the defect’s representation.

#### 2.3.4. Comparison with CAD Model

Measured datasets were also compared to the CAD model. Alignment was performed using the coordinate-system alignment command in the analysis software. In the next step, a pseudo-color surface-deviation map was created, which allows identification of misalignments or form deviations (e.g., flattening at the bottom of the sphere’s cavity). Since the measured part was faulty in general, this stage was only intended to evaluate the misalignment of the measured datasets.

## 3. Results and Discussion

**Figure 9:**
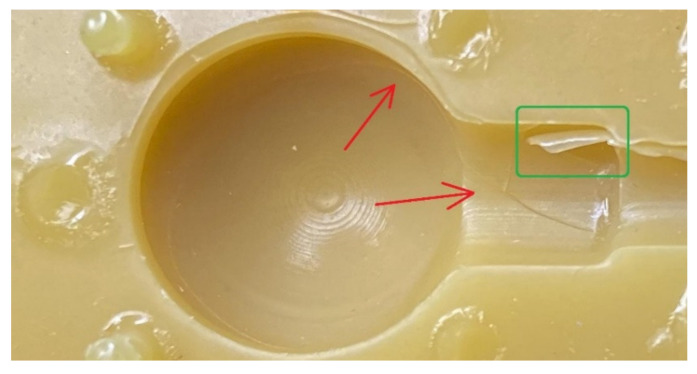
A magnified image of the printed male half of the die. Red arrows indicate cracks. The green box encompasses the flaking zone.

As stated before, the faulty element was deliberately chosen to test the systems’ ability to evaluate defects, e.g., cracks, bumps, and flaking. The investigated part was a 3D-printed injection die made of daylight flexible yellow resin (Figure 9). Numerous cracks on the parting plane, and one flaking zone, are visible with the naked eye. Cracks within the cavity are less visible and some may require the use of a magnifying glass.

### 3.1. Dimensional Accuracy

**Figure 10:**
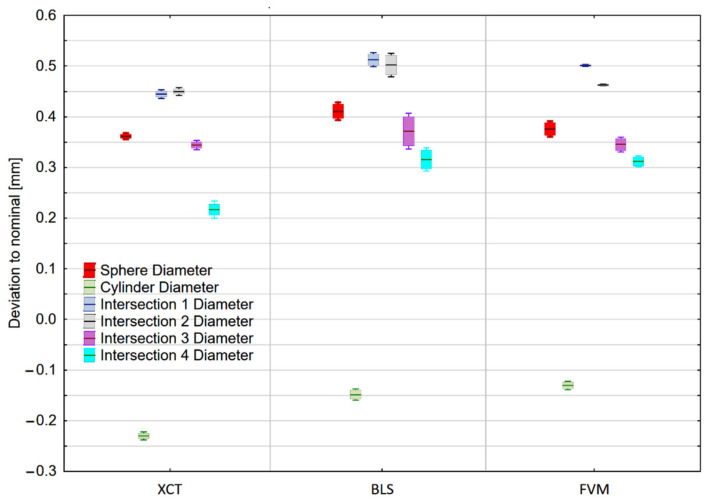
Measurement results of fitted geometric elements. Corresponding values of different elements are shifted for the purpose of data clarity. Box boundaries are equal to the standard deviation of the measurements, and whiskers represent the 95% confidence interval.

Mean values of the measured geometric elements and their standard deviations are presented in Figure 10.

None of the evaluated dimensions, except for the cylinder have met the tolerance limits. It appears that a major error occurred during the printing procedure, thus considering the deviation from the nominal value is irrelevant. Therefore, instead of unverifiable accuracy, the researchers focused on measurement reproducibility.

In the cylinder measurement the most reproducible measurements were performed with an X-ray CT device, achieving a standard deviation of 0.002 mm. The same device performed the most reproducible measurements of the sphere (standard deviation of 0.005 mm).

**Figure 11:**
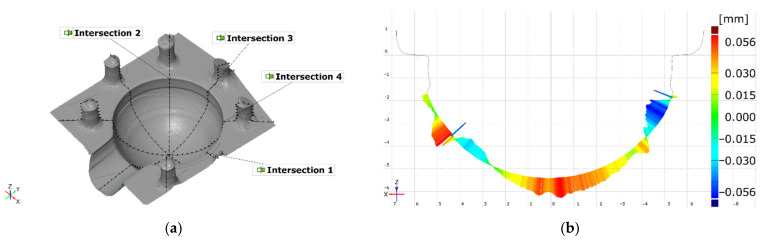
Visualization of the cross-sections and a fitted circle: (**a**) cross-sections as seen on the measured surface; (**b**) form deviations on a fitted circle on intersection 1.

Measurement of the fitted circles’ diameters (Figure 11 a,b) did not allow pointing out of the single most reproducible technology. In general, the highest values of standard deviations were recorded for the blue-light Scanner. Mean values of the measured characteristics can be found in Supplementary File—Tables S1–S3.

### 3.2. Form and Position Deviations

**Figure 12:**
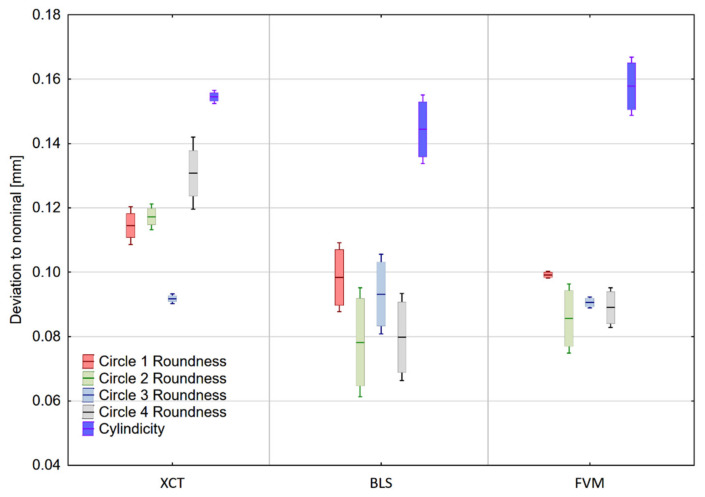
Values of roundness and cylindricity deviation on the measured sample. Corresponding values of different elements are shifted for the purpose of data clarity. Box boundaries are equal to the standard deviation of the measurements, and whiskers represent the 95% Confidence Interval.

As seen in Figure 12, the lowest values of standard deviation of the cylindricity measurement were recorded for the results from the XCT. This may be due to the most consistent representation of this feature, with no non-measured points and, therefore, most repeatable point selection for element creation. Other technologies achieved similar values of standard deviation. Both had significant amount of non-measured points prior to dataset preparation.

Measurement results of the fitted circles’ roundness were most consistent when measured with the focus variation microscope. Results from the XCT were most consistent in circles 1 and 3, and for circles 2 and 4 achieved values comparable to those from the blue-light Scanner. It must be noted, however, that each did not exceed the tolerance limits of 0.15 mm.

**Figure 13:**
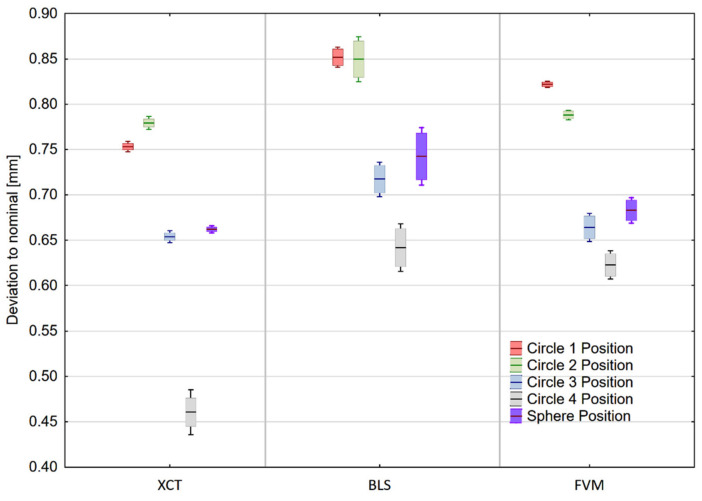
Positioning tolerances of fitted sphere and circles. Corresponding values of different elements are shifted for the purpose of data clarity. Box boundaries are equal to the standard deviation of the measurements, and whiskers represent the 95% Confidence Interval.

All geometric elements exceeded the maximum position tolerance value of 0.10 mm, as shown in Figure 13. This is most likely the outcome of the major printing error. The cross-section is shown in Figure 8b. Mean values of the measured characteristics can be found in Supplementary File—Tables S4–S9.

### 3.3. Comparison with CAD Model

**Figure 14:**
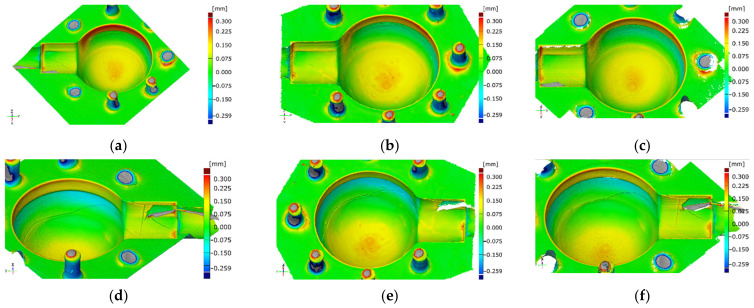
Surface comparison of measured element: (**a**) and (**d**)—XCT; (**b**) and (**e**)—BLS; (**c**) and (**f**)—FVM.

Surface comparison on aligned surfaces of both the actual data and the CAD model showed significant deviations in certain crucial areas. Firstly, the central area of the spherical cavity is elevated and flattened. The same observation can be recorded when analyzing cross section profiles (e.g., Figure 8b). Secondly, significant deformations can be seen in the proximity of the edges of the cylinder (Figure 14a–c). All measurement systems managed to detect the same artefacts on the cavity’s surface.

### 3.4. Defects Detection and Dataset Quality

**Figure 15:**
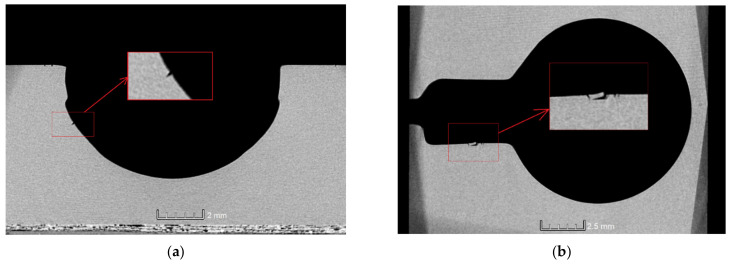
Two types of defects were present in the measured sample: (**a**) cracks; (**b**) flaking.

As stated before, the authors deliberately chose a faulty mold for this research purposes as a representation of possible defects and printing artefacts that may occur in the final product. These include: cracks, layers misalignment, bumps, and surface flaking (Figure 15). Overall dataset quality seems to be best in XCT measurements, where virtually no non-measured points exist. A significant amount of non-measured points was present in FVM measurements. However, it was the only method that was able to reproduce the printing layers. The blue-light scanner’s measurements resulted in a relatively smooth surface dataset that managed to reproduce some surface features, but in general, was not detailed enough for defects evaluation.

#### 3.4.1. Cracks

All measurement systems managed to detect major cracks on the element’s surface (Figure 14). However, only XCT and FVM featured a sufficient measurement’s resolution to reveal micro-scale defects (Figure 15a,b). XCT was also the only method that enabled the measurement of hidden surfaces (or re-entrant features [52]) and showed the extent of the cracks’ depth. The blue-light scanner failed to fully reproduce the cracks on the cylinder surface. However, they were clearly visible as defects.

#### 3.4.2. Top-Layer Flaking

**Figure 16:**
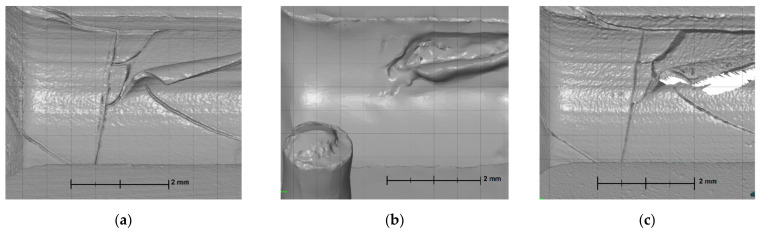
Flaking zone as measured with: (**a**) XCT; (**b**) BLS; (**c**) FVM.

The flaking effect occurred within the cylinder area and was initiated from the crack that encircles the entire cavity. Each measurement technology was able to measure the flake to some extent. However, it was only fully measured when using the XCT device (Figure 16a). The blue-light scanner was able to measure only a small part of the flake (Figure 16b), while the focus variation microscope (Figure 16c) required an additional measurement of the flake (Figure 6). The focus variation microscope was not able to measure the whole flake. However, it managed to provide a detailed view on most of the surface.

### 3.5. Results Summary

Main differences between the measurement results mainly originate from available resolution of used devices. In this study, the lowest resolution technique was the structured blue-light scanner, which yields maximum resolution of 0.02 mm. However, as it uses triangulation to obtain data, it may not detect cracks and crevices. Another limiting factor for both optical techniques (FVM and BLS) is varying surface reflectivity of the sample due to its curvature changes and the presence of small (submillimeter) radii on the edge of spherical cavity. In this study, the researchers used a semi-transparent resin, with a matt surface finish after curing. It is highly probable that it might have dispersed and/or absorbed the structured blue light, to an extent that resulted in lower quality of the scans. The aforementioned optical effects might also influence the quality of the data from FVM, since this technique relies on the contrast of the image. FVM, however, is capable of registering surfaces with high slope angles, in many cases up to 87 degrees, but in the case of the measured samples the light-dispersion effect was quite strong and lowered the quality of the measurement data on steep surfaces. Data quality was sufficient (i.e., little to no non-measured points or artefacts occurred) for surfaces with an inclination angle of up to 45–60 degrees. Both XCT and FVM could detect surface defects. However, the XCT scans yielded much better quality of the cracks and flaking geometry. XCT is unaffected by the optical characteristics of the sample, which allows for measurement of a variety of materials and surface textures. Moreover, X-ray imaging also enables to reliably measure internal structures and hidden or inaccessible surfaces, i.e., cracks and flaking, which is not possible when using optical techniques.

## 4. Conclusions

A DPP manufacturing method enables rapid and precise manufacturing of either prototypes or functional objects. In this study, the researchers evaluated three metrological systems for dimensional and quality evaluation of such element. All systems exhibited desired resolution (0.02 mm) of measurements. The maximum standard deviation of the measurement series was reported for circle 3 diameter, measured using BLS (0.028 mm). The highest values of standard deviations for XCT and FVM were recorded for cylinder and sphere diameter, respectively (0.017 mm and 0.012 mm). It must be noted, however, that the measurements performed with the blue-light scanner were overall the least reproducible, i.e., the standard deviation of the measured values was generally, with some exceptions, even several times higher, than in the other two systems. The choice of the right measurement system would be dictated by whether it is capable of both measuring within the demanded accuracy, and detecting any significant faults and artefacts that may occur during the printing process, such as flaking, cracks, cavities, etc. The most significant factor for the latter is the system’s resolution. In this research, each measurement system was able to detect artefacts smaller than 0.1 mm. The most sensitive system, regarding artefacts detection, is focus variation microscope, since it has the best resolution. This optical method is, however, highly susceptible to the resin material characteristic, i.e., its level of transparency and smoothness. There were multiple regions on the die’s surface that were locally smooth beyond the device’s capabilities (for the given objective) to detect any contrast on the surface. One way to overcome this problem would be to further increase the magnification, albeit at the cost of the measurement time.

The structured blue-light scanner was the fastest method of measurement, but yielded the lowest measurement resolution and failed to provide enough details to fully describe existing cracks, and the die’s edge on the parting plane. Although dimensional repeatability might improve when measuring larger parts, the device would still not be able to fully measure all surface flaws.

In the scope of further research, the most valuable measurement technique is XCT, since it allows not only measuring accurately and within a satisfying level of repeatability, but also is the only technique that is capable of measuring the inner surfaces, i.e., both cracks and the whole, closed die. Unlike the focus variation microscope, it is able to measure the whole object in one position, without the need of stitching separate measurements.

Non-measured points were most abundant in areas of the highest level of complexity (e.g., in the flaking zone), and steep edges or cracks. However, their presence does not have the same impact on the overall geometry as it would have on surface texture [53].

## Figures and Tables

**Table 1:** Measurement set-up for X-ray computed tomography.

Sensor Resolution/Voxel Size	X-ray Tube	No. of Projections	Exposure Time	Voltage	Current	Material Filter
1000 × 1000/15 μm	Transmission	1500	350 ms	120 kV	140 mA	none

**Table 2:** Measurements set-up for focus variation microscope.

Vertical Resolution	Lateral Resolution	Lens	Tilt	Illumination	Total Time
0.350 µm	8 µm	5×	45 degrees	Coaxial, polarized	28 min per single measurement

## Data Availability

Data available on request due to privacy restrictions.

## Supplementary Materials

The following are available online at https://www.mdpi.com/article/10.3390/ma14226758/s1, Table S1: Measurement of the fitted circles’ diameters made with XCT, Table S2: Measurement of the fitted circles’ diameters made with BLS, Table S3: Measurement of the fitted circles’ diameters made with FVM, Table S4: Position tolerance values measured with XCT, Table S5: Position tolerance values measured with BLS, Table S6: Position tolerance values measured with FVM, Table S7: Roundness and cylindricity deviation values measured with XCT, Table S8: Roundness and cylindricity deviation values measured with BLS, Table S9: Roundness and cylindricity deviation values measured with FVM.
