# Supplementary material for: Non-Contact Multiscale Analysis of a DPP 3D-Printed Injection Die for Investment Casting
Source: Materials (Basel). 2021 Nov 9;14(22):6758. doi: 10.3390/ma14226758 (PMC8624798; doi:10.3390/ma14226758)
Supplement: Supplementary file 1 [file materials-14-06758-s001.zip › materials-1419667-supplementary.pdf]

DIMENSIONS

Table S1

Value XCT

Feature	Nominal [mm]	Meas. 1	Meas. 2	Meas. 3	Meas. 4	Meas. 5	Mean	St.Dev
Sphere Dia.	11.18	0.353	0.357	0.36	0.364	0.365	0.3598	0.005
Cylinder Dia.	5	−0.193	−0.23	−0.229	−0.236	−0.224	−0.2224	0.017
Intersection 1 Dia.	11.18	0.428	0.441	0.44	0.446	0.452	0.4414	0.009
Intersection 2 Dia.	11.18	0.434	0.442	0.452	0.452	0.452	0.4464	0.008
Intersection 3 Dia.	11.18	0.331	0.337	0.343	0.345	0.351	0.3414	0.008
Intersection 4 Dia	11.18	0.207	0.215	0.217	0.204	0.23	0.2146	0.010

Table S2

Value Blue Light Scanner

Feature	Nominal [mm]	Meas. 1	Meas. 2	Meas. 3	Meas. 4	Meas. 5	Mean	St.Dev
Sphere Dia.	11.18	0.429	0.423	0.402	0.397	0.403	0.4108	0.014
Cylinder Dia.	5	−0.153	−0.136	−0.142	−0.158	−0.153	−0.1484	0.009
Intersection 1 Dia.	11.18	0.505	0.532	0.512	0.506	0.508	0.5126	0.011
Intersection 2 Dia.	11.18	0.509	0.529	0.502	0.479	0.492	0.5022	0.019
Intersection 3 Dia.	11.18	0.371	0.396	0.379	0.356	0.396	0.3796	0.017
Intersection 4 Dia	11.18	0.31	0.342	0.299	0.3	0.326	0.3154	0.018

Table S3

Value FVM

Feature	Nominal [mm]	Meas. 1	Meas. 2	Meas. 3	Meas. 4	Meas. 5	Mean	St.Dev
Sphere Dia.	11.18	0.382	0.383	0.378	0.382	0.354	0.3758	0.012
Cylinder Dia.	5	−0.133	−0.139	−0.129	−0.13	−0.121	−0.1304	0.007
Intersection 1 Dia.	11.18	0.5	0.502	0.502	0.499	0.502	0.501	0.001
Intersection 2 Dia.	11.18	0.462	0.461	0.465	0.462	0.463	0.4626	0.002
Intersection 3 Dia.	11.18	0.352	0.359	0.343	0.345	0.327	0.3452	0.012
Intersection 4 Dia	11.18	0.318	0.307	0.308	0.324	0.302	0.3118	0.009

POSITION

Table S4

Value XCT

Feature	Datum	Meas. 1	Meas. 2	Meas. 3	Meas. 4	Meas. 5	Mean	St.Dev
Circle 1 Position	Plane Z	0.751	0.751	0.75	0.754	0.758	0.753	0.003
Circle 2 Position	Plane Z	0.778	0.775	0.784	0.782	0.776	0.779	0.003
Circle 3 Position	Plane Z	0.659	0.648	0.655	0.654	0.658	0.655	0.004
Circle 4 Position	Plane Z	0.473	0.459	0.466	0.44	0.477	0.463	0.013
Sphere Position	Plane Z	0.668	0.659	0.662	0.665	0.662	0.663	0.003

Table S5

Value Blue Light Scanner

Feature	Datum	Meas. 1	Meas. 2	Meas. 3	Meas. 4	Meas. 5	Mean	St.Dev
Circle 1 Position	Plane Z	0.86	0.863	0.845	0.847	0.844	0.852	0.008
Circle 2 Position	Plane Z	0.875	0.866	0.842	0.829	0.836	0.850	0.018
Circle 3 Position	Plane Z	0.727	0.716	0.711	0.696	0.736	0.717	0.014
Circle 4 Position	Plane Z	0.66	0.664	0.617	0.624	0.644	0.642	0.019
Sphere Position	Plane Z	0.787	0.739	0.726	0.735	0.725	0.742	0.023

Table S6

Value FVM

Feature	Datum	Meas. 1	Meas. 2	Meas. 3	Meas. 4	Meas. 5	Mean	St.Dev
Circle 1 Position	Plane Z	0.819	0.823	0.823	0.819	0.825	0.822	0.002
Circle 2 Position	Plane Z	0.785	0.785	0.795	0.787	0.788	0.788	0.004
Circle 3 Position	Plane Z	0.669	0.68	0.66	0.665	0.646	0.664	0.011
Circle 4 Position	Plane Z	0.63	0.62	0.62	0.638	0.605	0.623	0.011
Sphere Position	Plane Z	0.687	0.69	0.684	0.69	0.663	0.683	0.010

FORM

Table S7

Value XCT

Feature	Meas. 1	Meas. 2	Meas. 3	Meas. 4	Meas. 5	Mean	St.Dev
Circle 1 Roundness	0.115	0.119	0.111	0.116	0.112	0.115	0.003
Circle 2 Roundness	0.11	0.117	0.118	0.12	0.114	0.116	0.003
Circle 3 Roundness	0.091	0.091	0.091	0.092	0.093	0.092	0.001
Circle 4 Roundness	0.123	0.129	0.128	0.141	0.125	0.129	0.006
Cylindricity	0.158	0.154	0.156	0.153	0.155	0.155	0.002

Table S8

Value Blue Light Scanner

Feature	Meas. 1	Meas. 2	Meas. 3	Meas. 4	Meas. 5	Mean	St.Dev
Circle 1 Roundness	0.089	0.103	0.101	0.109	0.09	0.101	0.008
Circle 2 Roundness	0.069	0.101	0.079	0.075	0.067	0.081	0.012
Circle 3 Roundness	0.088	0.094	0.089	0.11	0.085	0.095	0.009
Circle 4 Roundness	0.078	0.083	0.082	0.063	0.093	0.080	0.010
Cylindicity	0.149	0.155	0.144	0.132	0.142	0.143	0.008

Table S9

Value FVM

Feature	Meas. 1	Meas. 2	Meas. 3	Meas. 4	Meas. 5	Mean	St.Dev
Circle 1 Roundness	0.099	0.1	0.098	0.099	0.1	0.099	0.001
Circle 2 Roundness	0.08	0.083	0.101	0.082	0.082	0.087	0.008
Circle 3 Roundness	0.09	0.09	0.089	0.092	0.092	0.091	0.001
Circle 4 Roundness	0.084	0.097	0.09	0.087	0.087	0.090	0.004
Cylindricity	0.154	0.162	0.165	0.147	0.161	0.159	0.007
